# Retinal fluid quantification using a novel deep learning algorithm in patients treated with faricimab in the TRUCKEE study

**DOI:** 10.1038/s41433-024-03532-0

**Published:** 2024-12-11

**Authors:** Aamir A. Aziz, Arshad M. Khanani, Hannah Khan, Eileen Lauer, Ibrahim Khanani, Ohidul Mojumder, Zoha A. Khanani, Huma Khan, Greggory M. Gahn, J. Taylor Graff, Ashkan M. Abbey, David R. P. Almeida, Mark R. Barakat, Giulia Corradetti, Jordan M. Graff, Sara J. Haug, Jared S. Nielsen, Veeral S. Sheth, SriniVas R. Sadda, Ilan Hadas, Gidi Benyamini, Kester Nahen, Nishant Mohan

**Affiliations:** 1 University of Nevada, Reno School of Medicine, Reno, NV USA; 2 Sierra Eye Associates, Reno, NV USA; 3 Notal Vision, Manassas, VA USA; 4 Duke University, Durham, NC USA; 5 Midwestern University Arizona College of Osteopathic Medicine, Glendale, AZ USA; 6 Texas Retina Associates, Dallas, TX USA; 7 Erie Retinal Surgery, Erie, PA USA; 8 Retina Macula Institute of Arizona, Scottsdale, AZ USA; 9 Doheny Eye Institute, Los Angeles, CA USA; 10 Barnet Dulaney Perkins Eye Center, Phoenix, AZ USA; 11 Southwest Eye Consultants, Durango, CO USA; 12 Wolfe Eye Clinic, West Des Moines, IA USA; 13 University Retina, Chicago, IL USA; 14 Notal Vision, Tel Aviv, Israel

**Keywords:** Macular degeneration, Retinal diseases

## Abstract

**Background:**

Investigate retinal fluid changes via a novel deep-learning algorithm in real-world patients receiving faricimab for the treatment of neovascular age-related macular degeneration (nAMD).

**Methods:**

Multicenter, retrospective chart review and optical coherence tomography (OCT) image upload from participating sites was conducted on patients treated with faricimab for nAMD from February 2022 to January 2024. The Notal OCT Analyzer (NOA) algorithm provided intraretinal, subretinal and total retinal fluid for each scan. Results were segregated based on treatment history and fluid compartments, allowing for multiple cross-sections of evaluation.

**Results:**

A total of 521 eyes were included at baseline. The previous treatments prior to faricimab were aflibercept, ranibizumab, bevacizumab, or treatment-naive for 52.3%, 21.0%, 13.3%, and 11.2% of the eyes, respectively. Of all 521 eyes, 49.9% demonstrated fluid reduction after one injection of faricimab. The mean fluid reduction after one injection was −60.7nL. The proportion of eyes that saw reduction in fluid compared to baseline after second, third, fourth and fifth faricimab injections were 54.4%, 51.9%, 51.4% and 52.2%, respectively. The mean (SD) retreatment interval after second, third, fourth and fifth faricimab injection were 53.4 (34.3), 56.6 (36.0), 57.1 (35.3) and 61.5 (40.2) days, respectively.

**Conclusion:**

Deep-learning algorithms provide a novel tool for evaluating precise quantification of retinal fluid after treatment of nAMD with faricimab. Faricimab demonstrates reduction of retinal fluid in multiple groups after just one injection and sustains this response after multiple treatments, along with providing increases in treatment intervals between subsequent injections.

## Introduction

Neovascular age-related macular degeneration (nAMD), also known as wet or exudative AMD, is defined by pathological vasculature arising from the choriocapillaris, potentially damaging photoreceptors and causing permanent vision loss [1]. Retinal fluid as a result of exudation from these weakened vessels threatens vision by causing retinal pigment epithelium (RPE) detachment or subretinal fibrosis if untreated. Current and previous agents approved by the FDA to treat nAMD target vascular endothelial growth factor (VEGF), and include pegaptanib (OSI Pharmaceuticals, Long Island, NY, USA), ranibizumab (Genentech Inc, San Francisco, CA, USA), aflibercept 2 mg (Regeneron, Tarrytown, NJ, USA), brolucizumab (Novartis, Basel, Switzerland), faricimab (Genentech Inc, San Francisco, CA, USA), and aflibercept 8 mg (Regeneron, Tarrytown, NJ, USA) with the off-label use of bevacizumab (Genentech Inc, San Francisco, CA, USA) [2–6].

Faricimab was FDA approved in January 2022 for the treatment of nAMD and diabetic macular oedema (DMO). Faricimab is a bispecific, monoclonal antibody that targets both VEGF-A and angiopoetin-2 (Ang-2) and is intended to provide increased inhibition of neovascularization and exudation, along with greater durability between injections to address the high treatment burden experienced by patients [7, 8]. The Phase III studies, TENAYA and LUCERNE were two double-masked clinical trials comparing faricimab to aflibercept 2 mg [9]. Two year results demonstrated sustained, comparable improvements in visual acuity and retinal thickness between faricimab and aflibercept 2 mg, with greater durability for faricimab via the increased proportion of patients able to achieve 16- or 12-week intervals between one and two years and less frequent dosing required for similar efficacy [10]. The pooled post-hoc analysis from the TENAYA/LUCERNE studies also showed greater reduction in central subfield thickness (CST) and maximum pigment epithelial detachment (PED) thickness, improved fluid resolution, and greate resolution of serous PED with faricimab dual-inhibition compared to aflibercept 2 mg in the matched head-to-head dosing period [11]. These trials also demonstrated that the first absence of intraretinal fluid (IRF) and subretinal fluid (SRF) occurred quicker and with fewer injections in the faricimab arm compared to the aflibercept 2mg arm [12, 13].

The positive results from TENAYA and LUCERNE led to the FDA approval of faricimab but are limited by their demographic of only treatment-naïve patients. Real-world studies are not bound by this limitation and can investigate the effect of faricimab on patients who have received injections previously, prior to making a switch. This allows for the efficacy of faricimab to be investigated in patients with persistent disease after prolonged prior treatment, patients who could not be extended on prior anti-VEGF agents or difficult-to-treat naive patients with subretinal haemorrhages or extensive PED who were not included in the Phase 3 clinical trials. Prior-treated patients with active disease and high treatment burden can help us evaluate the efficacy of a new medication, especially those with an additional mechanism of action. It is here that faricimab functions as an additional tool for clinicians by simultaneously targeting a novel pathway of Ang-2 inhibition while also treating the primarily implicated pathogenesis driven by VEGF.

The presence of retinal fluid on optical coherence tomography (OCT) is a key biomarker of disease activity that is used by physicians for the management of nAMD. In different treatment regimens like *pro re nata* (PRN) and treat-and-extend, retinal fluid presence on OCT scans is used to further personalize treatment decisions and timing of the next treatment, along with changes in agents to find the most efficacious drug in controlling retinal fluid with the greatest durability to improve vision outcomes, compliance and patient satisfaction [14, 15].

Several clinical trials have used retinal fluid as a key metric for defining retreatment criteria and/or as an endpoint to evaluate efficacy of a therapeutic strategy. Comparison of AMD Treatments Trial (CATT) used presence of retinal fluid as one of the criterions for treating patients in the PRN arm of the study [16]. The change in retinal thickness or central subfield thickness (CST), the average thickness over the central 1-mm of the macula, is often used as a proxy for quantifying the amount of the fluid present in the retina as it can be quickly obtained from OCT. Pivotal trials for nAMD treatments such as ranibizumab, aflibercept and faricimab used changes in CST as a secondary endpoint, including TENAYA and LUCERNE, which also used change in CST as a criterion for retreatment in the investigational arms.

However, change in CST has limitations when used as a substitute for the amount of fluid present in the retina. Change in CST is a less sensitive measure as it provides the average change over an existing baseline value. The smaller amounts of fluid may be obscured by the measurement variance of much larger thickness values. By definition, CST evaluates the central 1-mm of the macula which mostly excludes influence from any fluid present outside this region. Thickness changes can also occur for reasons other than fluid presence, such as diffuse retinal thickness or subretinal fibrosis. Additionally, the same volume of fluid can cause different amounts of thickness changes dependent on distribution of fluid volume pockets in the retina. Finally, the retinal thickness change value alone does not characterize if the change was due to intraretinal or subretinal fluid, or potentially a combination of both. This distinction is particularly crucial as a number of studies have shown differences in disease prognosis and outcomes depending on the dominant fluid type for a given eye [17].

These limitations have led to interest in developing algorithms for directly quantifying the retinal fluid volume as demonstrated in OCT images. Recent efforts have particularly gained momentum with the latest developments in artificial intelligence using machine learning and deep learning-based methods to segment fluid pixels present in the intraretinal and subretinal spaces. Classical machine learning methods that use hand crafted features have been used for segmenting the fluid in the retina, with the first clinical results published in 2016 [18]. However, the most favourable methods have used deep neural networks, particularly the U-net architecture [19, 20]. The efficiency of U-net architecture in image segmentation stems from precise representation of higher order information while preserving the context through connections between earlier and later stages of the neural network.

The purpose of this sub-analysis from the TRUCKEE study is to evaluate precise anatomic response of dual inhibition of VEGF-A and Ang-2 with faricimab in a real-world patient population by utilizing a deep learning algorithm to quantify retinal fluid.

## Methods

### Participants

Patients from nine sites (Sierra Eye Associates, Reno, NV; Texas Retina Associates, Dallas, TX; Erie Retinal Surgery, Erie, PA; Retina Macula Institute of Arizona, Scottsdale, AZ;. Doheny Eye Institute, Los Angeles, CA; Barnet Dulaney Perkins Eye Center, Phoenix, AZ; Southwest Eye Consultants, Durango, CO; Wolfe Eye Clinic, West Des Moines, IA; University Retina, Chicago, IL) were identified based on the inclusion criteria of receiving faricimab for the treatment of nAMD. It was determined by the Advarra Institutional Review Board (IRB) that the study is exempt from IRB oversight as no patient-identifying information is collected. Confidentiality was maintained at individual sites to ensure that no shared data or data aggregate would include any identifying information. All patients who received faricimab for the treatment of nAMD were included and no excluding criteria were placed to additionally filter subjects, making this a true real-world analysis.

Spectralis (Heidelberg Engineering, Heidelberg, Germany) OCT scans acquired from the nine participating sites were uploaded to a central secure server. All OCT scans were composed of 49 B-scans covering a 6 mm × 6 mm transverse area. All OCT volume scans were processed using the Notal OCT Analyzer (NOA, Notal Vision, Tel Aviv, Israel) volume. The algorithm provided intraretinal, subretinal and total retinal fluid volumes for each scan. The algorithm also provided a fluid thickness map corresponding to intraretinal, subretinal and total retinal fluid. The NOA algorithm has been validated in other clinical studies and has shown to provide comparable results to the manual segmentation provided by expert graders [21]. A version of this algorithm used for home-based optical coherence tomography (OCT) was recently approved by the US Food and Drug Administration (FDA).

### Fluid quantification using Notal OCT Analyzer

The Notal OCT Analyzer (NOA) utilizes an artificial intelligence (AI) algorithm to automatically detect and quantify retinal fluid, encompassing both IRF and SRF, in Spectralis, Cirrus and Notal Vision Home OCT scans. Employing deep learning, an advanced machine learning technique, the algorithm engages in semantic segmentation. This process involves the computer algorithm learning the mapping from images to class labels using extensive labelled training data, where each pixel in an image is categorized into classes including retinal/nonretinal tissue, IRF and SRF. The NOA’s deep learning architecture involves a convolutional neural network (CNN) that first performs segmentation and subsequently quantifies retinal fluid. Specifically, a modified U-net-based CNN module is used for segmentation of the retina at the internal limiting membrane and retinal pigment epithelium layers, along with separate segmentation of IRF and SRF. A second module then classifies these segmented regions, and the retinal fluid volume is then quantified independently for IRF and SRF. The algorithm has demonstrated highly accurate segmentation and quantification of retinal fluid [22, 23]. These findings contributed to the establishment of the nanolitre (nL) as a recommended unit of measure for retinal fluid [24]. In addition to quantification, the NOA generates annotated B-scans, depicting areas of retinal fluid color-coded by fluid type, enface maps of fluid thickness (individually for IRF and SRF), and a ranking of B-scans based on the order of largest to smallest fluid area.

### Fluid change after 1st faricimab treatment

The number of eyes that demonstrated fluid reduction after the 1st faricimab treatment was recorded. The mean retreatment interval of both the prior agent and from baseline to the 1st faricimab treatment was recorded. In order to eliminate very small fluid fluctuations counting towards increasing or decreasing fluid, the percentage of eyes that saw reduction in fluid and had at least 10-nL of fluid in one of the visits were recorded. The retreatment interval for these eyes was also recorded. The mean (SD) and median (IQR) reduction in fluid for all eyes was recorded. A distribution of overall fluid change and retreatment interval for all eyes was calculated. The above parameters were separately calculated for the cohorts of the eyes that were previously treated with aflibercept and the eyes that were treatment naive. This additional analysis allows to understand switch outcomes for the most commonly used agent aflibercept and also helps elucidate outcomes for the treatment naive patients that are expected to demonstrate significantly different disease dynamics.

### Fluid change after multiple faricimab treatments

The number of eyes that demonstrated reduction in fluid after the first, second, third, fourth and fifth faricimab treatments were recorded. The mean (SD) retreatment interval after the second, third, fourth and fifth faricimab injections were recorded. In order to eliminate recording very small amounts of fluctuations as counting towards increasing or decreasing fluid, the percentage of eyes that saw reduction in fluid and had at least 10-nL of fluid in one of the visits was recorded. The above parameters were separately calculated for the cohorts of the eyes that were previously treated with aflibercept and the eyes that were treatment naive.

### Role of fluid compartments

Fluid dynamics were analysed for the eyes based on presence of fluid in different compartments. The percentage of eyes with only IRF at baseline, only SRF at baseline, and with both IRF and SRF at baseline that saw fluid reduction after the first, second, third, fourth and fifth treatment were recorded. The mean (SD) and median (IQR) change in fluid for eyes with different compartments at successive faricimab treatments was recorded. Considering only the eyes that had fluid greater than 10-nL at least once, the number of eyes with fluid reduction after the second, third, fourth and fifth faricimab injections were recorded.

## Results

All eyes that had complete data uploaded and at least 2 scans were analysed. A total of 521 eyes were included at baseline. The median (IQR) age for the included patients was 80.7 (11) years. Of these patients, 56.0% of patients were female. The previous treatments before switching to faricimab were aflibercept, ranibizumab, bevacizumab, brolucizumab, treatment-naive and others for 48.2%, 18.5%, 11.8%, 5.3%, 9.9% and 6.3% of the eyes respectively.

### Fluid change after 1st faricimab treatment

**Fig. 1:**
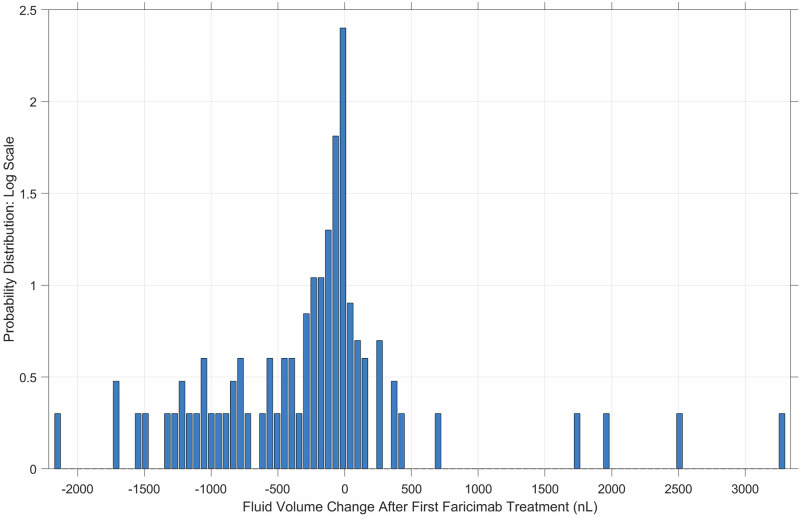
The distribution of the amount of fluid change after the first faricimab treatment. The distribution is plotted on a log scale for easy visualization of smaller values. The distribution demonstrates long tails with a high proportion of eyes showing decrease in fluid amounts compared to those showing an increase in fluid.

A total of 49.9% (260 of 521) of eyes saw fluid reduction after the first faricimab treatment. It should be noted that 32% (171 of 521) of eyes did not demonstrate fluid at the time of first treatment. The mean (SD) retreatment intervals for these eyes before and after the first faricimab treatment was 44.4 (28.2) and 53.0 (35.5) days, respectively. When considering only the eyes with greater than 10-nL of fluid at either visit (those with meaningful fluid manifestation), 69.8% (180 of 258) of eyes saw reduction in fluid. The mean fluid reduction for all eyes was −60.7 nL. A distribution of overall fluid change is shown in Fig. 1.

Among eyes with last treatment as aflibercept 2 mg (*n* = 296), 50.0% (148 of 296) demonstrated fluid reduction. The mean retreatment intervals for these eyes before and after the first faricimab treatment was 44.9 (26.5) and 49.3 (29.9) days respectively. For eyes with greater than 10-nL of fluid at either visit, 67.6% (96 of 142) saw fluid reduction. The mean reduction for all eyes previously treated with aflibercept was −57.0 nL.

Among the treatment-naive eyes (*n* = 49), 81.6% (40 of 49) demonstrated fluid reduction. The mean retreatment intervals for these eyes after the first faricimab treatment was 41.8 days. For eyes with greater than 10-nL of fluid at either visit, 91.2% (31 of 34) saw fluid reduction. The mean fluid reduction for all naive eyes was −219.2 nL.

### Fluid change after multiple faricimab treatments

**Fig. 2:**
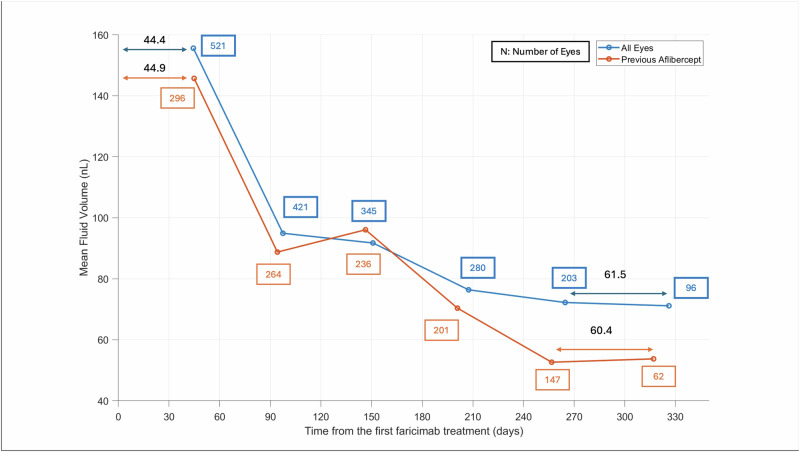
The mean fluid change after successive faricimab treatments for all eyes and eyes that were previously treated with aflibercept. We see a decline in mean fluid volumes after the first and successive faricimab treatments, along with an increase in the treatment interval after multiple injections of faricimab.

The mean fluid changes for all eyes observed from baseline to after the 5th faricimab treatment are shown in Fig. 2. The results for successive treatments in all eyes are summarized in Table 1a, where a stable proportion of patients with fluid reduction is maintained and extension of treatment intervals are demonstrated.

**Table 1:** **a** Fluid reduction and retreatment interval for all eyes and all eyes with fluid greater than 10-nL after successive treatments of faricimab. We see a stable proportion of all eyes near 50% for improvements in total retinal fluid with increases in treatment intervals. In all eyes with fluid greater than 10-nL, we see over 60% of patients demonstrating improvement in total retinal fluid with increases in treatment intervals; **b** Fluid reduction and retreatment interval for all eyes previously treated with aflibercept after successive treatments of faricimab. We see a stable proportion of eyes above 50% for improvements in total retinal fluid with increases in treatment intervals. In eyes with fluid greater than 10-nL, we see over 57% of patients demonstrating improvement in total retinal fluid with increases in treatment intervals; **c** Fluid reduction and retreatment interval for all treatment-naive eyes after successive treatments of faricimab. We see a stable proportion of eyes above 80% for improvements in total retinal fluid. In eyes with fluid greater than 10-nL, we see over 85% of patients demonstrating improvement in total retinal fluid.

(a) All Eyes						
After Treatment #	Eyes (*n*/*N*)	All Eyes		Eyes (*n*/*N*)	Eyes with > 10-nL Fluid	
		% with fluid reduction	Mean Retreatment Interval (days)		% with fluid reduction	Mean Retreatment Intervals (days)
1	260/521	49.9%	53.0	180/258	69.8%	49.5
2	229/421	54.4%	53.4	169/224	75.5%	56.6
3	179/345	51.9%	56.6	135/199	67.8%	55.5
4	144/280	51.4%	57.1	112/169	66.3%	54.3
5	106/203	52.2%	61.5	81/135	60.1%	62.9

(b) Previously Treated with Aflibercept						
After Treatment #	Eyes (*n*/*N*)	All Eyes		Eyes (*n*/*N*)	Eyes with > 10-nL Fluid	
		% with fluid reduction	Mean Retreatment Interval (days)		% with fluid reduction	Mean Retreatment Intervals (days)
1	148/296	50.0%	49.3	96/142	67.6%	49.6
2	136/264	51.5%	52.2	93/131	71.0%	50.7
3	121/236	51.3%	54.5	89/137	65.0%	51.4
4	103/201	51.4%	55.8	77/116	66.4%	54.1
5	77/147	52.2%	60.4	53/92	57.6%	58.7

(c) Treatment-naive						
After Treatment #	Eyes (*n*/*N*)	All Eyes		Eyes (*n*/*N*)	Eyes with > 10-nL Fluid	
		% with fluid reduction	Mean Retreatment Interval (days)		% with fluid reduction	Mean Retreatment Intervals (days)
1	40/49	81.6%	41.8	31/34	91.2%	38.1
2	34/37	91.9%	39.2	28/29	96.6%	64.2
3	21/26	80.8%	55.3	17/20	85.0%	71.3
4	16/21	76.2%	62.8	14/16	87.5%	54.7
5	13/15	86.7%	51.3	12/14	85.7%	67.3

The mean fluid changes from baseline to after the 5th faricimab treatment for all eyes with aflibercept 2 mg as the last treatment prior to receiving faricimab are shown in Fig. 2. The results for successive treatments in all eyes previously treated with aflibercept are summarized in Table 1b, where a stable proportion of patients with fluid reduction is maintained and extension of treatment intervals are demonstrated.

**Fig. 3:**
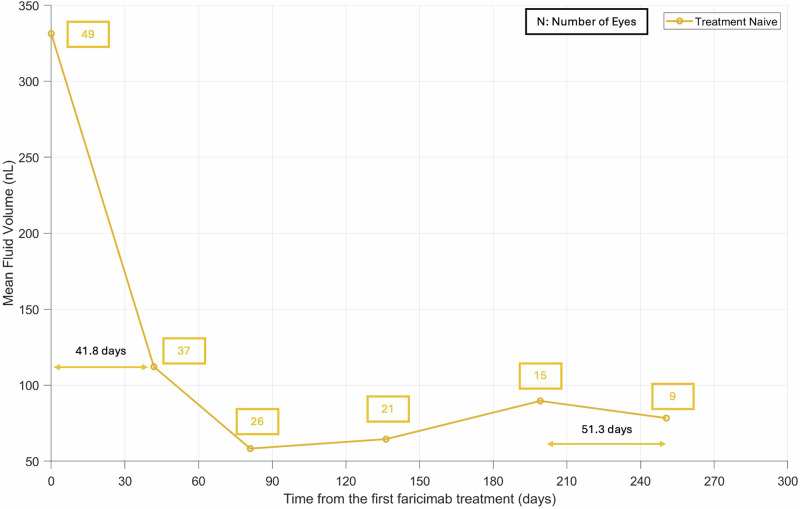
The mean fluid change after successive faricimab treatments for treatment naive eyes. We see a decline in mean fluid volumes after the first and successive faricimab treatments, along with an increase in the treatment interval after multiple injections of faricimab.

The mean fluid changes for all treatment-naive eyes from baseline to after the 5th faricimab treatment are shown in Fig. 3. The results for successive treatments in all treatment-naive eyes are summarized in Table 1c, where a large proportion of patients with fluid reduction is maintained and extension of treatment intervals are demonstrated after multiple injections.

### Role of fluid compartments

At baseline, among the eyes that had at least 2 faricimab treatments, 123 had both IRF and SRF, 104 of eyes presented IRF only, and 123 of eyes presented SRF only. A total 171 eyes had no fluid manifestation at baseline.

#### Both IRF and SRF at baseline

Among eyes that only had both IRF and SRF present at baseline, a fluid reduction was demonstrated in greater than 80% of patients, which remained consistent for the successive four treatments. The treatment intervals for these patients also remained stable. Full results are available within Table 2.

**Table 2:** Fluid reduction and retreatment interval for all eyes stratified by retinal fluid compartments.

After Treatment #	Eyes (*n*/*N*)	All Eyes with IRF & SRF		Eyes (*n*/*N*)	Eyes with > 10-nL Fluid	
		% with fluid reduction	Mean Retreatment Interval (days)		% with fluid reduction	Mean Retreatment Intervals (days)
1	100/123	81.3%	54.3	89/109	81.7%	52.6
2	83/100	83.0%	57.6	76/91	83.5%	50.4
3	68/81	84.0%	57.5	63/74	85.1%	58.6
4	52/61	85.3%	53.7	47/56	83.9%	53.2
5	33/39	84.6%	62.6	30/36	83.3%	64.7

After Treatment #	Eyes (*n*/*N*)	All Eyes with IRF only		Eyes (*n*/*N*)	Eyes with > 10-nL Fluid	
		% with fluid reduction	Mean Retreatment Interval (days)		% with fluid reduction	Mean Retreatment Intervals (days)
1	66/104	63.5%	51.9	29/50	58.0%	47.0
2	64/82	77.1%	48.5	34/46	73.9%	44.6
3	50/70	71.4%	53.4	29/39	65.9%	48.0
4	35/55	63.5%	56.4	22/37	59.5%	54.8
5	32/45	71.1%	53.1	19/32	59.4%	51.7

After Treatment #	Eyes (*n*/*N*)	All Eyes with SRF only		Eyes (*n*/*N*)	Eyes with > 10-nL Fluid	
		% with fluid reduction	Mean Retreatment Interval (days)		% with fluid reduction	Mean Retreatment Intervals (days)
1	94/123	76.4%	50.7	62/87	71.3%	47.9
2	82/103	79.6%	53.9	59/73	80.8%	54.3
3	61/81	75.3%	56.2	43/60	71.7%	48.0
4	57/68	83.8%	55.1	43/53	81.1%	55.6
5	41/51	80.4%	60.7	31/41	75.6%	59.7

We see a stable proportion of over 80% of eyes with both IRF and SRF demonstrating improvements in total retinal fluid. Eyes with IRF only demonstrate a proportion greater than 60% of patients with improvement. Eyes with SRF only demonstrate a proportion greater than 75% of patients with improvement.

#### IRF only at baseline

Among eyes that only had IRF present at baseline, a fluid reduction was demonstrated in greater than 60% of patients, which remained consistent for the successive four treatments. The treatment intervals for these patients also remained stable. Full results are available within Table 2.

#### SRF only at baseline

Among eyes that only had SRF present at baseline, a fluid reduction was demonstrated in greater than 75% of patients, which remained consistent for the successive four treatments. The treatment intervals for these patients also remained stable. Full results are available within Table 2.

## Discussion

Real-world data has become critical to the evaluation of newly approved medications, a key component in investigating the clinically relevant utilization of new medications. Clinical trials create controlled environments for treatment and outcomes but the resulting limitations set by inclusion/exclusion criteria can create difficulty in assessing generalizability of results. Comprehensive real-world studies allow for different patient populations to be investigated beyond the scope of clinical trials, but are inherently limited by a lack of resources typically devoted to a randomized trial and many times, as in this case, by their retrospective nature. However, when taken together, data from clinical trials and real-world investigations help to complement each other and overcome the different sets of limitations typical of each.

Previously published results from the TRUCKEE study demonstrated a statistically significant improvement in both visual acuity and anatomy via CST and fluid reduction, along with comparable safety to that of the Phase III trials [25]. Other real-world studies, including case series, retrospective and prospective studies have demonstrated similar findings, highlighting the positive effects of dual-inhibition of both VEGF-A and Ang-2 [26–31]. Results from TRUCKEE and other studies also included decreases in the proportion of patients with retinal fluid, subdivided as IRF or SRF. However, presence of retinal fluid was evaluated in a binary fashion, limiting investigation of fluid improvements to only the number of patients with total resolution, as determined by individual investigators. Effectively, patients who demonstrated significant and clinically meaningful reductions in their fluid volume without full resolution are not represented by this value. By utilizing the NOA, not only are images standardized with consistent calculations of fluid volume, but reduction in volume burden can be precisely calculated.

Fluid reduction is demonstrated by NOA calculation, where we see that reduction occurs in a majority of patients with meaningful fluid burden (>10-nL), whether previously-treated or treatment-naive, after just one injection of faricimab. Baseline characteristics of all eyes calculate a total retinal fluid volume of nearly 155.6-nL, with one injection of faricimab reducing fluid volume by an average of 60.7-nL, nearly a 40% decrease with a single injection. Sustained reductions of total retinal fluid continued with successive treatments of faricimab, an average decrease of 85.5-nL after five treatments.

These findings are consistent even in subgroups divided by treatment history, demonstrating anatomic efficacy of faricimab in patients previously treated with aflibercept, the current gold-standard agent, including those that may be considered “difficult-to-treat” or “high-need” by their physicians. Even with a lower potential for anatomic improvement in these patients, a rapid and sustained decrease in retinal fluid volume is seen with just one injection of faricimab and maintained with subsequent injections. Similarly, treatment-naive patients had a decrease of over 200-nL after just one injection of faricimab, and stayed below 100-nL of total retinal fluid with continued therapy to five treatments. Interestingly, the effect of dual-inhibition of VEGF-A and Ang-2 revealed a unique pattern of improving SRF in a higher proportion of patients than IRF, especially those with the predetermined value of greater than 10-nL of total retinal fluid. In patients with both IRF and SRF, a high rate of patients with improvements in retinal fluid volume are seen.

Ultimately, faricimab continues to demonstrate efficacy in improving anatomic parameters for all subdivisions of patients while also increasing treatment interval in many of these patients. Deep learning algorithms such as the NOA are powerful tools for studying the anatomic benefits of new medications, by standardizing image reads and providing precise measurements instead of binary data collection. Further data will continue investigating the effect of faricimab on retinal fluid, as illuminated by the NOA to provide detailed and consistent quantification of these parameters. In addition, we intend to investigate the effect of fluid volumes, fluid compartments and transverse localization of the fluid on visual acuity outcomes after switching to faricimab.

## Summary

### What was known before

- Faricimab is a novel, bispecific monoclonal antibody that inhibits VEGF-A and Ang-2 for neovascular AMD, and has been shown to have efficacy in visual acuity and anatomy with comparable safety and increased durability.

### What this study adds

- This real-world study utilizing a deep learning algorithm quantifies retinal fluid to demonstrate retinal fluid resolution and improvement at a minute level.
